# Effect of *Berchemia discolor* Leaf Meal (Muni Tree) on Feed Intake, Growth Performance and Digestibility of Non-Descript Goats

**DOI:** 10.3390/ani15091342

**Published:** 2025-05-06

**Authors:** Jobere Anastacia Mashiachidi, Tlou Grace Manyelo, Busisiwe Gunya

**Affiliations:** Department of Agricultural Economics and Animal Production, University of Limpopo, Polokwane 0727, South Africa; 201918876@keyaka.ul.ac.za (J.A.M.); grace.manyelo@ul.ac.za (T.G.M.)

**Keywords:** caprine, digestibility, feed conversion, intake, leaf meal supplementation

## Abstract

Seasonal feed shortages often limit goat productivity in rural areas, creating a need for alternative, locally available feed resources. This study evaluated the effect of *Berchemia discolor* leaf meal on feed intake, digestibility and growth performance of South African non-descript goats. Sixteen yearling goats (average age 12 months, body weight 19.63 ± 1.68 kg) were randomly assigned to four dietary treatments in a completely randomized design and housed individually in metabolic cages. They were fed diets containing 0%, 15%, 20% and 30% *B. discolor* leaf meal, formulated on a dry matter basis for 42 days (14-day adaptation, 28-day measurement). *B. discolor* supplementation had no effect on weight and feed intake (*p* > 0.05). However, goats fed diets with 0% and 30% inclusion levels had a significantly higher average daily gain and more efficient feed conversion ratio compared to those fed 15% and 20% inclusion levels (*p* < 0.05). Daily dry matter intake did not differ (*p* > 0.05) among groups. However, *B. discolor* had lower (*p* < 0.05) crude protein (CP), neutral detergent fiber (NDF) and acid detergent fiber (ADF) contents compared to the control (0% inclusion). Metabolic weight intakes were similar (*p* > 0.05) across treatments. *B. discolor* had no effect (*p* > 0.05) on dry matter digestibility, but CP and ADF digestibility were higher (*p* < 0.05) in goats fed 0% *B. discolor* than those in 15%, 20% or 30% inclusion groups. In conclusion, *B. discolor* inclusion improved growth performance and feed conversion efficiency in non-descript goats, though higher levels reduced nutrient digestibility. Inclusion up to 20% supported intake and growth, but the highest feed conversion ratio was observed at 0% and 30% inclusion.

## Simple Summary

Goat farming plays an important role in rural livelihoods, but seasonal feed shortages often limit productivity. This study evaluated the effect of *Berchemia discolor* leaf meal on the growth performance and nutrient digestibility of South African non-descript goats. Sixteen yearling South African non-descript goats (averaging 12 months old, body weight 19.63 ± 1.68 kg) were randomly assigned to four dietary treatments in a completely randomized design and fed experimental diets containing 0%, 15%, 20% and 30% *Berchemia discolor* leaf meal, formulated on a dry matter basis for 42 days. Feed intake and body weight remained similar across all groups, but weight gain and feed conversion improved with supplementation. The diets influenced crude protein and fiber content, with crude protein decreasing slightly at higher inclusion levels due to tannin presence, while neutral detergent fiber and acid detergent fiber increased with more leaf meal due to higher fiber content in *B. discolor*. Despite reduced digestibility at 15% and 30% inclusion levels, *B. discolor* leaf meal showed potential as a valuable feed supplement because it is rich in crude protein and supported growth when used at 0% or 30% inclusion levels, where feed efficiency was highest. It can help improve goat nutrition, especially during dry seasons when quality feed is scarce. These findings offer practical options for communal farmers to enhance goat productivity and food security using locally available resources.

## 1. Introduction

Goat farming in South Africa is expanding due to the increasing demand for goat products driven by population growth [1]. Goats are well adapted to diverse environments, demonstrating their resilience to dry conditions and high temperatures. However, in many tropical and subtropical regions, the productivity of small ruminants, particularly goats, remains low due to various constraints, including diseases, poor management practices and limited access to high-quality feed, especially during the prolonged dry season [2].

Seasonal fluctuations in pasture availability and quality significantly affect ruminant nutrition, often leading to nutrient deficiencies [3]. Research has consistently shown that South African non-descript goats experience weight loss and deteriorating body condition during the dry season [4,5]. Therefore, identifying alternative nutrient-rich feed resources is essential to mitigate these challenges and enhance goat productivity. *Berchemia discolor* leaves have been identified as a promising feed, containing approximately 20.02% crude protein, 32.45% neutral detergent fibre and 20.87% acid detergent fibre, making them a valuable source of nutrients for goats. Globally, various unconventional alternative feed resources have been used to address these challenges, including marginal drought-resistant fodders, algae [6,7] insects [8] and industrial byproducts [9].

Browse tree legumes and shrub foliage play a crucial role in providing fodder for livestock in communal rangelands of Southern Africa. One of the key benefits of browse trees is their ability to improve feed intake and growth performance when supplemented with fibrous basal diets [10]. *Berchemia discolor* has gained attention as a potential feed supplement for goats, particularly in regions facing nutritional challenges. Its leaves are rich in protein, making them a valuable source of nutrients essential for goat health and productivity [11]. Several studies have demonstrated that *Berchemia discolor* leaf meal can help address nutrient deficiencies commonly observed in goats, especially in communal areas during the dry season [12,13,14].

Evaluating the impact of *B. discolor* on goat nutrition is essential for determining its effectiveness as a dietary supplement. This study assessed both growth performance and digestibility parameters to understand how *B. discolor* inclusion influences nutrient utilization and overall animal productivity. The objective of this study was to determine the effect of *B. discolor* leaf meal on the nutrient intake, digestibility and growth performance of South African non-descript goats. It was hypothesized that the inclusion of *B. discolor* leaf meal in goat diets would improve growth performance and feed conversion efficiency, but higher inclusion levels may reduce nutrient digestibility due to the presence of tannins.

## 2. Materials and Methods

This study was conducted during spring (from August to September 2024), at the University of Limpopo Experimental Farm Syferkuil, Limpopo Province, South Africa. Practice for care and use of animals for experimental purposes were reviewed and approved by the Ethics Committee on the Use of Animal for Research, certificate number AREC/12/2024: PG.

### 2.1. Experimental Design and Animal Management

Sixteen yearling intact male South African non-descript goats with an initial body weight of 19.63 ± 1.68 kg were used in this experiment. Male South African non-descript goats were acclimatized to the experimental diet for 2 weeks to observe their health status.

Following the adaptation period, a relative preference study was conducted with a cafeteria feeding approach, as described by Larbi et al. [15], using the treatment diets. Goats were raised in individual metabolic cages (1.5 × 0.8 m) equipped with a feeder and drinker separately to allow them to move around freely and lie down.

The goats were randomly assigned to 4 dietary treatment levels (0%, 15%, 20% and 30% *B. discolor* leaf meal inclusion) using a completely randomized design (CRD), with 4 goats per treatment group, each serving as a replicate. The inclusion levels of *B. discolor* leaf meal were formulated on a dry matter basis. No vaccinations or deworming treatments were administered during the adaptation period, as all goats were clinically healthy at the start of the experiment.

A compound feed was mixed with *B. discolor* leaves, grass hay (*Cenchrus ciliaris*) and lucerne pellets (Table 1). The inclusion of *B. discolor* leaf meal in the goats’ diet was calculated on a dry matter basis. The treatment diets were formulated to contain 0%, 15%, 20% and 30% of *B. discolor* leaf meal. The ingredients, including lucerne pellets and buffalo grass (*Cenchrus ciliaris*), were all adjusted on a dry matter basis to ensure accurate nutrient intake. The lucerne pellets were commercially purchased from Angel feeds in Polokwane, South Africa. The buffalo grass was harvested, dried and chopped before being incorporated into the diets.

This study was conducted in 4 stages, namely preparation, adaptation, treatment and data collection periods. The preparation stage lasted for a week and involved setting up the pens, in addition to selecting and tagging the goats. The experiment lasted for 42 days, including a 14-day adaptation period followed by 28 days of data collection. The pens were cleaned daily by the chief researcher, who also helped in monitoring goats daily. Feeding troughs were cleaned before being filled with a fresh diet to ensure that the new diet was always clean and free of fungus. The goats were provided with individual diets and ad libitum clean water.

### 2.2. Growth Performance

At the commencement of this study, goats were weighed individually using the SellEton SL-930 Portable Livestock Animal weighing scale (2000 lb capacity, digital display), which offers precise weight measurements and portable design to measure changes in body weight in response to the experimental treatments. Thereafter, goats were weighed weekly, with final body weight measurements taken on the last day of the study period. Live weight was used to calculate the growth rate of the goats.

The average daily gain (ADG) was calculated by dividing the total weight gain by the number of days in the trial (28 days). The daily amount of feed provided and the morning feed refusal per goat were measured using an electronic weighing balance (AE ADAM), with the difference between these values recorded to compute the daily feed intake.

A surplus margin of approximately 10% was maintained daily to allow voluntary feed intake and ensure ad libitum consumption. Goats were provided with the diet every morning at 8:30 am, once every day until the last day of the study. The average daily feed intake (ADFI) was then calculated by averaging the total feed consumed over 28 days. Feed conversion ratio was calculated as the summation amount of feed ingested divided by the live weight gain of the goats.

### 2.3. Digestibility Trial

Apparent nutrient digestibility measurements were performed in metabolic cages equipped with independent watering and feeding troughs. Each goat was fitted with a green faecal collection bag (Biobag) for in vivo digestibility. The goats were adapted for 3 days to the carrying of faecal collection bags, which was followed by a total faecal collection for 3 successive days for each animal.

At the end of the collection period, the faeces of individual animals were thoroughly mixed, and a sample was taken for chemical analysis. Faecal samples were oven-dried at 60 degrees Celsius for 48 h and ground to pass through a 1-mm sieve prior to chemical analysis. The apparent digestibility coefficient of crude protein, neutral detergent fibre and acid detergent fibre were calculated based on nutrient intake and faecal output, following the method described by [16].

$$\mathrm{Nutrient}\;\mathrm{Digestibility}=\frac{Nutrient\;intake-faecal\;nutrient\;output}{Nutrient\;intake}\times 100$$

### 2.4. Chemical Analysis

The samples were subjected to chemical analysis to determine dry matter (DM) and crude protein (CP) content following the standard methods of the Association of Official Analytical Chemists (AOAC) at the University of Pretoria, Animal Nutrition Lab, South Africa. Dry matter was determined by oven drying at 105 °C using a Memmert UFB 400 oven until a constant weight was achieved, according to AOAC 930.15.

Crude protein content was analyzed using the Kjeldahl method with a Buchi K-370 distillation unit, as described in AOAC 984.13. Furthermore, acid detergent fibre (ADF) and neutral detergent fibre (NDF) were determined according to the methods of Van Soest et al., as cited in reference [17]. The nutrient composition of *B. discolor* leaf meal comparable to the nutrients required by goats are shown in Table 2.

### 2.5. Statistical Analysis

Growth performance data were analyzed using one-way analysis of variance (ANOVA) in the Statistical Package for the Social Sciences SPSS, 2020. Where significant differences (*p* < 0.05) were detected, Duncan’s Multiple Range Test was employed to compare treatment means. This test was chosen for its suitability in identifying pairwise differences among multiple treatments commonly used in animal nutrition studies. The *p*-value was considered significantly different at 95% interval (*p* <0.05).

Data on diet intake, nutrient intake and in vivo digestibility of South African non-descript goats were analysed using the General Linear Model procedures in SAS Version 9.4 (2012). Where significant effects were detected, treatment means were separated using Fisher’s least significant difference (LSD) test. Differences were considered statistically significant at *p* < 0.05.

The data were analyzed using the following model:

$$Y_{ij}=\mu +T_{i}+e_{ij}$$

Yij = the observed value in the portion that received the treatment i in repetition j;

μ = overall mean;

Ti = the fixed effect of the inclusion level of B. bicolor (i = 0, 15, 20 and 30 g/kg);

eij = random error, normal and independently distributed with a mean of zero and variance of δ2 (NID)~(0, σ2).

## 3. Results

### 3.1. Nutrient Digestibility

The results of the effect of *Berchemia discolor* leaf meal inclusion level on diet intake (g/goat/day), nutrient intake (g/kgW^−0.75^) and digestibility in South African non-descript goats are presented in Table 3.

*B. discolor* leaf meal inclusion level had no effect (*p* > 0.05) on daily dry matter intake. However, *B. discolor* leaf meal inclusion level affected (*p* < 0.05) diet crude protein (CP), neutral detergent fibre (NDF) and acid detergent fibre (ADF) contents.

The diet having a 0% *B. discolor* leaf meal inclusion level had a higher (*p* < 0.05) CP content than those having a 15 or 30% *B. discolor* leaf meal inclusion level. However, the CP diet having a 20% *B. discolor* leaf meal inclusion level was similar (*p* > 0.05) to a diet having a 0% *B. discolor* leaf meal inclusion level. Similarly, CP diet having a 20% *B. discolor* leaf meal inclusion level had no effect (*p* > 0.05) on diets containing a 15 and 30% *B. discolor* leaf meal inclusion level.

Diet NDF and ADF values were higher (*p* < 0.05) in goats consuming a diet having a 30% *B. discolor* leaf meal inclusion level as compared to diets containing 0 or 15% *B. discolor* leaf meal inclusion levels. Similarly, NDF intake values were higher (*p* < 0.05) in goats fed diets having a 20% *B. discolor* leaf meal inclusion level than those having a 0 or 15% *B. discolor* leaf meal inclusion level.

Goats consuming diets containing a 0 or 15% *B. discolor* leaf meal inclusion level had similar (*p* > 0.05) NDF intakes. Goats on a diet having a 20% *B. discolor* leaf meal inclusion level had a significantly higher (*p* < 0.05) ADF compared to those on a diet having a 0% *B. discolor* leaf meal inclusion level. However, no significant differences (*p* > 0.05) were observed on ADF diet having 20 and 15% *B. discolor* leaf meal inclusion levels. Similarly, goats on a diet containing 0 and 15% *B. discolor* leaf meal inclusion levels were similar (*p* > 0.05).

Intakes per metabolic weight of goats were similar (*p* > 0.05) across the treatments, ranging from 42.68 to 39.44 g/kgW^−0.75^ of dry matter. Similarly, goats consumed the same (*p* > 0.05) amount of crude protein (CP), neutral detergent fibre (NDF) and acid detergent fibre (ADF) contents. Diet CP, NDF and ADF digestibility values were significantly different (*p* < 0.05) across the treatments. However, no significant differences (*p* > 0.05) were observed on diet DM digestibility.

Goats on a diet having a 0% *B. discolor* leaf meal inclusion level had higher (*p* < 0.05) CP and ADF digestibility than those on a diet containing 15, 20 or 30% *B. discolor* leaf meal inclusion levels. NDF digestibility decreased (*p* < 0.05) with an increased *B. discolor* leaf meal inclusion level.

### 3.2. Growth Performance

The results of the effect of *B. discolor* leaf meal inclusion level on the liveweights, feed intake, average daily gain (ADG) and feed conversion ratio (FCR) of South African yearling non-descript goats are presented in Table 4. The inclusion of *B. discolor* leaf meal had no effect (*p* = 0.853) on initial live weight, (*p* = 0.569) live weight, (*p* = 0.665) final live weight and (*p*= 0.205) feed intake.

However, significant differences were observed in (*p* = 0.009) average daily gain (ADG) and (*p* = 0.029) feed conversion ratio (FCR), with goats on a 0% *B. discolor* leaf meal inclusion diet showing the highest ADG and the best FCR, compared to those on 15% and 20% inclusion levels. Goats on a diet with 0% *B. discolor* leaf meal inclusion had a significantly higher (*p* = 0.009) ADG (158.3g) compared to those on a diet with 15% (83.3g) or 20% (83.3g) inclusion.

However, the average daily gain of goats on diets containing 0% or 30% *B. discolor* leaf meal inclusion were not significantly different (*p* > 0.05). Similarly, goats on diets containing 15% and 20% *B. discolor* leaf meal inclusion levels had similar (*p* > 0.05) ADG; there were no significant differences in the ADG between diets containing 15% and 20% *B. discolor* leaf meal.

Goats on a diet containing a 0% *B. discolor* leaf meal inclusion level had a better (*p* = 0.029) FCR value compared to those on diets with 15% or 20% *B. discolor* leaf meal inclusion levels. Similarly, goats on a diet with a 30% *B. discolor* leaf meal inclusion level had a better (*p* = 0.029) FCR value than goats on a diet with 15% and 20% *B. discolor* leaf meal inclusion levels. However, goats on a diet having 15% and 20% *B. discolor* leaf meal inclusion levels had similar (*p* > 0.05) FCR values. Similarly, goats on a diet having 0% or 30% *B. discolor* leaf meal inclusion levels had similar (*p* > 0.05) FCR values.

## 4. Discussion

### 4.1. Nutrient Digestibility

*Berchemia discolor* leaf meal inclusion levels of 0 to 30% in the present study had no significant effect on dry matter (DM) and nutrient intake per metabolic weight of the goats. These findings are consistent with the previous reports [18,19,20], which reported that moderate levels of 1–4% of condensed tannins in the diet from different plant sources exerted no significant effect on the diet intake. Contrarily, [21] there were observed significant differences between the control and supplemented groups in daily total DM intake per unit of metabolic weight and as per cent of body weight. These could be because of differences in body weight changes and feed utilisation efficiency among the experimental animals. In the current study, the similarity in DM intake across treatments suggests that goats were able to adapt to the diets due to moderate tannin levels that did not exert strong anti-nutritional effects.

The diets containing 0 and 20% *B. discolor* leaf meal inclusion levels provided 177 and 162.15 g/goat/day CP contents, respectively. The higher CP contents in these diets were due to the protein contribution from *B. discolor* inclusion levels, indicating that diets with up to 20% inclusion could meet maintenance requirements and support some production levels in ruminants. This finding is consistent with the results reported by [22]. However, the results of the current study are contrary to the results that were observed by [23], who documented that the protein supplemented diets had the greatest CP content followed by the *Acacia* leaf meal diets, with the rangeland hay diet (control) having the least.

Fibre is crucial for proper rumen function and its digestibility affects overall feed efficiency. As expected, nitrogen detergent fibre and acid detergent fibre contents were higher in control diets than in those containing *B. discolor* leaf meal, which aligns with the general trend that plant-based diets tend to have higher NDF than ADF contents. Previous studies [24,25] have reported that high fibre content limits DM intake and digestibility, whereas diets with lower levels of the fibre are associated with high voluntary DM intakes, as observed in this study. The increased feed intake at a 30% *B. discolor* leaf meal inclusion level could be attributed to reduced structural fibre components, making the diet more palatable and easier to digest.

The results of the current study showed that the inclusion levels of *B. discolor* leaf meal in the diet affected the digestibility of CP, NDF and ADF. Diet control resulted in higher CP digestibility compared to the supplemented diets due to the absence of tannins, which are known to form complexes with proteins and reduce their availability for digestion [26]. The reduction in CP digestibility at higher *B. discolor* levels suggests that condensed tannins interfered with ruminal protein degradation and microbial activity, limiting nitrogen utilization. The present results disagree with the observations of [27], who documented that the CP digestibility value increased with an increasing proportion of the diets.

Fibre (NDF and ADF) digestibility declined with increasing *B. discolor* inclusion due to the presence of tannins and lignified fibre components that reduce microbial access to structural carbohydrates. Tannins can form complexes with proteins and carbohydrates, limiting their availability to rumen microbes and inhibiting microbial enzymes responsible for fiber breakdown [28]. This trend is consistent with previous findings by [29], who reported that high tannin levels negatively impacted fibre digestion by inhibiting microbial enzymes responsible for fibre breakdown.

However, [30] found no significant differences in detergent fibre digestibility among supplemented diets, which may be due to variations in tannin tolerance among animal species or adaptation mechanisms over time. The observed decline in fibre digestibility in this study suggests that higher *B. discolor* inclusion levels may compromise nutrient absorption and overall digestive efficiency. Tannins can alter ruminal fermentation efficiency by affecting microbial populations and enzyme activities, leading to reduced fiber degradation. While goats possess some adaptive mechanisms to tannins, the extent of adaptation may depend on the duration of exposure and the specific tannin profile of the diet [31]. Further investigations into the long-term adaptation responses in goats to tannin-rich diets are warranted to optimize dietary formulations and improve nutrient utilization.

### 4.2. Growth Performance

According to [32], *Berchemia* species are considered cost-effective sources of protein and can be included in goat diets as leaf meals. In this present study, the inclusion of *B. discolor* leaf meal at 0-30% had no significant effect on feed intake per goat and feed intake per metabolic weight of the goats. These findings align with previous reports by [24,33], which noted that basal diet intake declined with supplementation, particularly at 15% inclusion (T2). The lack of a significant difference in feed intake among treatments may be attributed to the good palatability of *B. discolor* leaf meal, as goats in all treatments readily consumed the diets. Similarly, [21] observed that basal diet was higher in the control treatment compared to the leaf-supplemented group, although a non-significant difference was observed among the supplement’s groups. This suggests that supplementation reduces basal diet intake.

Despite the lack of significant differences in live weight and feed intake, average daily gain (ADG) varied significantly among treatments. This discrepancy could be due to differences in nutrient utilization efficiency, influenced by the balance of protein and energy or anti-nutritional factors such as tannins. In the present study, the goats attained a positive growth rate in treatment 1 and 4, reflected in desirable live weight gains, and remained in good health condition throughout the experiment. This may indicate that treatment 2 and 3 did not provide an optimal balance of nutrients for growth due to fibre content affecting nutrient digestibility or protein–energy imbalances.

The observed reduction in basal diet intake at treatment 2 and 3 suggests that tannin content may have negatively affected overall palatability or voluntary intake at those levels. This reflects the complex interaction between palatability and anti-nutritional factors like tannins. These findings align with a recent meta-analysis by [34], which evaluated the impacts of dietary tannins in small ruminants. Their study concluded that while moderate tannin levels may improve nitrogen retention and antioxidant status, high levels can impair growth performance, fibre digestibility and ruminal fermentation efficiency. This supports the notion that the negative effects observed at intermediate inclusion levels in this present study are due to excessive tannin intake. Thus, variations in growth response to *B. discolor* leaf meal could be attributed to the dual nature of tannins, which offer some benefits at low levels while inhibiting feed utilization and nutrient absorption at higher levels.

The feed conversion ratio (FCR) further supports this pattern. Goats fed diets with 0% and 30% *B. discolor* leaf meal inclusion levels demonstrated better feed conversion efficiency compared to those fed intermediate inclusion levels (15% and 20%). This suggests that at intermediate levels, *B. discolor* leaf meal may have limited nutrient bioavailability or an imbalanced nutrient profile, affecting weight gain efficiency. These findings are consistent with [35] but contrast with [36], who reported a significant difference in FCR between the control and supplemented diets. One explanation is that indigenous goats, as noted by [37], efficiently utilize local forage, which may influence how they respond to different dietary treatments.

The reduced efficiency observed at intermediate inclusion levels could be attributed to several factors. An imbalance in the energy-to-protein ratio may impair nutrient utilization, leading to suboptimal growth performance. Moreover, the presence of condensed tannins in *B. discolor* can bind to dietary proteins and carbohydrates, forming complexes that reduce their digestibility and availability. This binding effect may also interfere with ruminal microbial activity, further compromising nutrient absorption. Additionally, tannins can affect gut microbiota composition, potentially altering fermentation patterns and nutrient metabolism. These combined effects may contribute to the observed decrease in feed conversion efficiency at intermediate inclusion levels.

## 5. Conclusions

*B. discolor* inclusion up to 20% supported nutrient intake and growth, while 30% inclusion also improved feed conversion efficiency. However, digestibility was compromised at 15% and 30% inclusion levels. Therefore, up to 20% inclusion is recommended to balance intake, growth and digestibility in South African non-descript goats. These findings suggest that *B. discolor* can be used in goat diets without negatively affecting short-term performance. Further research is needed to assess its long-term impact on growth, digestion and potential rumen adaptation to tannin.

## Figures and Tables

**Table 1:** Composition of feed materials in the experimental diets.

Feed	Co	BFL_15_	BFL_20_	BFL_30_
*Berchemia discolor* leaf (%)	0	15	20	30
Hay (*Cenchrus ciliaris*) (%)	66	51	46	36
Lucerne pellets (%)	34	34	34	34
Total (%)	100	100	100	100

Co: control diet. BFL: *Berchemia discolor* leaf meal.

**Table 2:** Chemical composition of *B. discolor*.

Nutrients	Contents (%)
DM	79.97
CP	20.02
NDF	32.45
ADF	20.87

DM: dry matter; CP: crude protein; NDF: nitrogen detergent fibre, ADF: acid detergent fibre.

**Table 3:** Effect of different inclusion levels of *B. discolor* leaf meal on diet intake, nutrient intake and digestibility of South African non-descript goats.

		Treatments				
Variables	Co	BFL_15_	BFL_20_	BFL_30_	SEM	*p*-Value
	Diet Intake (g/goat/day)					
DM	850.33	807.66	883.45	901.04	23.809	0.185
CP	177.50 ^a^	146.31 ^b^	162.15 ^ab^	147.03 ^b^	4.499	0.006
NDF	367.25 ^b^	364.47 ^b^	429.07 ^a^	481.20 ^a^	10.799	0.001
ADF	191.16 ^c^	214.61 ^bc^	243.76 ^ab^	272.62 ^a^	6.088	<0.001
	Nutrient Intake (g/kgW^−0.75^)					
DM	39.44	43.06	41.89	42.68	3.979	0.922
CP	8.24	7.80	7.69	6.97	0.737	0.702
NDF	17.03	19.43	20.35	22.79	1.869	0.246
ADF	8.87	11.44	11.56	12.91	1.052	0.110
	Digestibility (%)					
DM	88.88	88.27	89.38	89.56	0.342	0.233
CP	43.56 ^a^	32.59 ^c^	40.03 ^b^	26.88 ^d^	0.010	<0.001
NDF	78.06 ^a^	65.16 ^b^	61.66 ^c^	31.92 ^d^	0.009	<0.001
ADF	51.97 ^a^	41.89 ^c^	43.97 ^b^	33.77 ^d^	0.079	<0.001

a,b,c,d: means with different superscripts in the same row are significantly different (*p* < 0.05). BFL: *Berchemia discolor* leaf meal. DM: dry matter; CP: crude protein; NDF: neutral detergent fibre; ADF: acid detergent fibre. SEM: standard error of mean. Co: control diet.

**Table 4:** Effect of *B. discolor* leaf meal on growth performance of South African non-descript goats.

Attributes	Treatments					
	Co	BFL_15_	BFL_20_	BFL_30_	SEM	*p*-Value
Initial weight (kg)	19.5	18.4	20.3	20.3	0.824	0.853
Liveweight (kg/goat/day)	22.7	19.7	21.7	22.3	0.769	0.569
Final weight (kg)	22.9	20.1	22.1	23.0	0.862	0.665
Feed intake (g/goat/day)	941.7	890.5	977.3	988.1	17.737	0.205
ADG (g/goat/day)	158.3 ^b^	83.3 ^a^	83.3 ^a^	129.8 ^b^	10.602	0.009
FCR (g/g)	6.1 ^a^	12.3 ^b^	12.1 ^b^	7.9 ^ab^	0.859	0.029

a, b: means in the same row not sharing the same superscript are significantly different (*p* > 0.05). BFL: *Berchemia discolor* leaf meal. FCR: Feed Conversion Ratio; ADG: Average Daily Gain. SEM: standard error of mean. Co: control diet.

## Data Availability

Dataset available on request from the authors.
